# Social Transmission of Avoidance Behavior under Situational Change in Learned and Unlearned Rats

**DOI:** 10.1371/journal.pone.0006794

**Published:** 2009-08-27

**Authors:** Akira Masuda, Shuji Aou

**Affiliations:** Department of Brain Science and Engineering, Kyushu Institute of Technology, Kitakyushu, Japan; Medical College of Georgia, United States of America

## Abstract

**Background:**

Rats receive information from other conspecifics by observation or other types of social interaction. Such social interaction may contribute to the effective adaptation to changes of environment such as situational switching. Learning to avoid dangerous places or objects rapidly occurs with even a single conditioning session, and the conditioned memory tends to be sustained over long periods. The avoidance is important for adaptation, but the details of the conditions under which the social transmission of avoidance is formed are unknown. We demonstrate that the previous experience of avoidance learning is important for the formation of behaviors for social transmission of avoidance and that the experienced rats adapt to a change of situation determined by the presence or absence of aversive stimuli. We systematically investigated social influence on avoidance behavior using a passive avoidance test in a light/dark two-compartment apparatus.

**Methodology/Principal Findings:**

Rats were divided into two groups, one receiving foot shocks and another with no aversive experience in a dark compartment. Experienced and inexperienced rats were further divided into subjects and partners. In Experiment 1, each subject experienced (1) interaction with an experienced partner, (2) interaction with an inexperienced partner, or (3) no interaction. In Experiment 2, each subject experienced interaction with a partner that received a shock. The entering latency to a light compartment was measured. The avoidance behavior of experienced rats was inhibited by interaction with inexperienced or experienced partners in a safely-changed situation. The avoidance of experienced rats was reinstated in a dangerously-changed situation by interaction with shocked rats. In contrast, the inexperienced rats were not affected by any social circumstances.

**Conclusions/Significance:**

These results suggest that transmitted information among rats can be updated under a situational change and that the previous experience is crucial for social enhancement and inhibition of avoidance behavior in rats.

## Introduction

Various social animals interact with conspecifics and use information from other animals to adapt to their environments. The transmission of information by interaction or observation is called social transmission. Social transmission is shaped by social clues, which consist of visual, olfactory, acoustic, or other types of information from conspecifics. Many studies have shown that social interaction or simple observation of other animals' behavior has significant effects on food preference [1]–[3], acquisition of motor patterns [4]–[6], and avoidance [7]–[9] in many species of vertebrate including primates, birds, fish, and rodents (for a review, see [10]). Rats, one of the most common experimental animals, prefer to ingest the same type of food as that ingested recently by a conspecific [2], [11]. This social transmission of food preference is thought to be formed by an association between food odorants and a volatile component of a rat's breath [12].

One of the most important behaviors affecting survival is the avoidance of dangerous objects or places. Avoidance learning is formed through an operant-conditioning process. In passive avoidance, for example, animals are punished for entering a preferred place by a footshock, and then the animals stop entering the place. This learning also includes some aspects of Pavlovian-conditioning [13]–[14]. In avoidance learning, association between an aversive stimulus and the environmental context (and its components) also can be shaped. Some previous studies reported that rats did not learn avoidances socially [15]–[16]. For example, rats do not learn avoidance just by watching conspecifics receiving a shock [17] or by interaction with poisoned conspecifics [14]. Other paper showed that rats learned to avoid a candle flame by exposure to another rat acquiring the same avoidance responses [18]. These conflicting results probably come from the different experimental conditions.

One possible factor is subjects' experience. The various responses following social interaction could be affected by the responder's experience. For example, social recognition requires semantic memories and knowledge obtained previously by experiences [19]. The perception of another's pain, and empathy for pain, are dependent upon bottom-up factors (i.e., observation of another person's pain expression and contextual pain cues) as well as top-down factors (i.e., features of the observer's own experience of pain and knowledge) (for a review, see [20]).

A recent study has shown that rats, like humans, can apply previous learning to adapt to new situations [21]. Social transmission of food preference also interacts previous learning in rats [22]. Therefore, experience of individual learning should be important for various perceptions and decision making even by rats. In the previous studies concerning social transmission of avoidance, many researchers used naïve rats as subject animals. Considering that not only social cues but also subjects' experience are important for social recognition, we believe one possible explanation why rats did not learn avoidance socially could be that the association between top-down factors (avoiding experience of individuals) and bottom-up factors (social clues from others) was not formed because naïve rats have no experiences of pain or another aversive stimulus.

Adaptive behavior learned in response to a dynamic environment is surely determined by the changing conditions of the environmental situation. There is dynamic interaction between the learning of avoidance behavior and a situation. Avoidance behavior is adaptive in an environment that includes a danger, but this behavior will be discontinued if the danger disappears. Social influence has the potential to improve the adaptation to an environment with a situational change, because the probability of receiving a signal of danger or safety as well as the possibility of sharing the signal change becomes high in social conditions. However, the effect of social influence on adaptation to a change of situation, especially from danger to safety or safety to danger, is not known, while that of adaptation to a novel situation has been investigated in detail. In the present study we focused on subjects' experience of avoidance learning and investigated uncertain dynamics, that is, the social influence on avoidance behavior in response to a situational change. We conducted two sequential experiments. In Experiment 1, we examined the effect of social interaction on avoidance behavior in a safely-changed situation where the shock stimulus was lost. In Experiment 2, we examined the social influence in a dangerously-changed situation where the shock stimulus was renewed.

## Materials and Methods

### Subjects

The subjects were 77 male Wistar rats aged 8 weeks, acquired from Kyudo Co., Ltd. (Kumamoto, Japan). They were given free access to food and water, and housed two per cage for one week before the start of the experiments. Housing conditions were thermostatically controlled at 22–24°C with a light/dark cycle (lights on: 08:00—20:00). The experiments were performed under the control of the Ethics Committee of Animal Care and Experimentation in accordance with the Guiding Principles for Animal Care Experimentation, Kyushu Institute of Technology, Japan, and with the Japanese Law for Animal Welfare and Care.

### Apparatus

**Figure 1:**
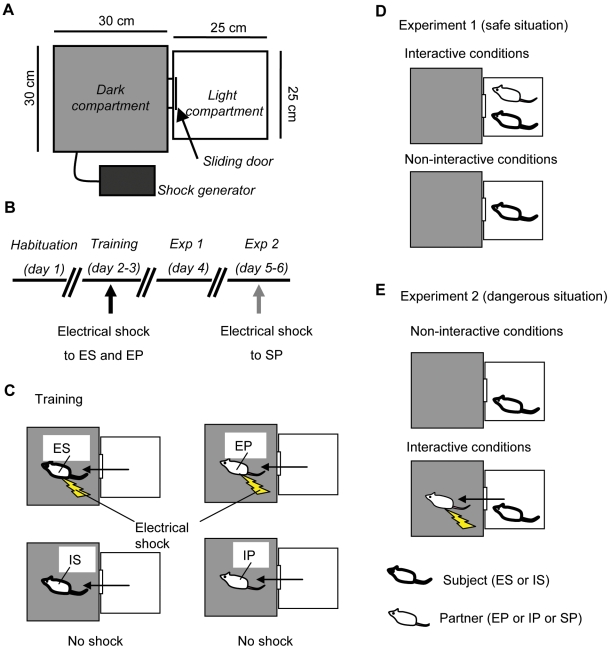
The experimental design. (A) Experimental apparatus. (B) Time schedule of this study. The black arrow shows electric shock to the experienced subjects and partners (ES: experienced subjects; IS: inexperienced subjects; EP: experienced partners; IS: inexperienced partners). The gray arrow shows electric shock to the partners (SP: shocked partners). (C–E) Overview of the experiments. (C) The schematic diagram of the training session. The left row indicates the treatment for the subjects (ES: experienced subjects; IS: inexperienced subjects); the right row indicates the treatment for the partners (EP: experienced partners; IP: inexperienced partners). (D) The schematic diagram of Experiment 1. The upper row indicates interactive conditions, and the lower row indicates non-interactive conditions. (E) The schematic diagram of Experiment 2. The upper row shows non-interactive conditions, and the lower row shows interactive conditions.

The experiments took place in a test chamber consisting of two compartments, a light compartment (D25 cm×W25 cm×H27 cm) and a dark compartment (D30 cm×W30 cm×H30 cm) (Figure 1A). The two compartments were divided by a sliding door. Electric shocks are delivered by a shock generator (SGS-002, Muromachi Kikai Co., Ltd., Tokyo, Japan). In Experiment 2, a removable partition was used to prevent subject animals from moving from one compartment to the next earlier than the partners.

### Procedure

All treatments or behavioral tests were done during the light cycle (12:00–20:00) in the following sequence (the whole schedule is shown in Figure 1B):

#### 1. Training

On the first day of this session (day 1), all animals were placed in the light compartment for 1 min individually and habituated to the experimental apparatus. After this interval, the sliding door was raised and the latency to enter the dark compartment was recorded. On the second day (day 2), a single electrical shock (0.5 mA, 5 s) was induced inescapably on 40 animals in the dark room after each animal entered the dark compartment, and they were used as the experienced subjects and 5 partners. The other 37 animals who received no electrical shocks were used as inexperienced subjects and partners. The experimental apparatus was cleaned with alcohol to remove odors before treating the next subject. On the third day (day 3), the latency of each animal to enter the dark compartment was measured. The schematic diagram of the training is shown in Figure 1C.

#### 2. Experiment 1

The subjects were divided into three groups: i) together with an experienced partner (EP), ii) with an inexperienced partner (IP), iii) without any partner (No). On the day following the training session (day 4), each subject was placed in the light compartment. If partnered, they were paired with the partner rats for 1 min. After the interval, the sliding door was raised and then the latencies to enter the dark compartment were measured, with a cut-off time of 15 min. This experiment was performed without any electric shocks. The schematic diagram of Experiment 1 is shown in Figure 1D.

#### 3. Experiment 2

The day after Experiment 1 was performed (day 5), experienced and inexperienced subjects were put in the experimental apparatus individually and habituated to the dark compartment for 20 min. On the second day of this experiment (day 6), 30 min before the test trial, each animal was placed in the light compartment and then the latencies to enter the dark compartment were measured, with a cut-off time of 5 min. We used the experienced subjects that entered within a given cut-off time as the experienced subjects (n = 16) and randomly selected inexperienced subjects (n = 12). In a test trial, each subject was placed in the light compartment with a partner for 1 min. Then, the sliding door was raised to permit the partners only to enter the dark compartment. During this time, a mesh partition attached in the center of the light compartment (in between a subject and a partner) did not permit the subjects to enter the dark compartment. After the partner entered, electrical shocks (0.5 mA, 3–6 s) were induced. Immediately after that, the partner returned to the light compartment and stayed there. After an additional interval (30 s), the partition was removed. The latencies to enter the dark compartment were measured with a cut-off time (15 min). The partner rat stayed in the light compartment and could interact with the subject freely during the measurement. We then compared the latency between the two conditions, with no partner and with a shocked partner. The schematic diagram of Experiment 2 is shown in Figure 1E. All partners were the rats already used in Experiment 1, which had been given a single foot shock to stabilize partners' pain reaction (habituation to the shock).

### Data analysis

Data were analyzed with the use of SPSS software (version 16.0). Before analysis, the Kolmogorov-Smirnow test was performed for normality. In Experiment 1, we used the Turkey-Kramer multiple comparison test to assess the statistical significance of the difference among the rat groups. In Experiment 2, we used a paired *t*-test to evaluate the statistical significance of the difference between measurements in the absence and presence of partners. The criterion for statistical significance was *p*<0.05 (two-tailed).

## Results

### Social interaction on avoidance behavior in a safe situation

For preparation, we trained 40 rats individually (30 subjects and 10 partners) to avoid the dark room by using electrical stimuli (0.5 mA, 5 s), and the other 37 rats (28 subjects and 9 partners) did not receive the training. The trained rats and untrained rats were used as experienced rats and inexperienced rats, respectively. We examined the influences of social interaction on the avoidance behaviors in a safe situation under the following 6 conditions: i) experienced subjects with inexperienced partners (ES-IP), ii) experienced subjects with experienced partners (ES-EP), iii) experienced subjects without any partners (ES-No), iv) inexperienced subjects with inexperienced partners (IS-IP), v) inexperienced subjects with experienced partners (IS-EP), (vi) inexperienced subjects without any partners (IS-No). As a summary, all combinations are presented in Table 1.

**Table 1:** The conditions for Experiment 1 and Experiment 2.

Condition	Interactive	Subject	Partner
Experiment 1			
(i) ES-IP	YES	Experienced	Inexperienced
(ii) ES-EP	YES	Experienced	Experienced
(iii) ES-No	NO	Experienced	(-)
(iv) IS-IP	YES	Inexperienced	Inexperienced
(v) IS-EP	YES	Inexperienced	Experienced
(vi) IS-No	NO	Inexperienced	(-)
Experiment 2			
ES-No	NO	Experienced	(-)
ES-SP	YES	Experienced	Shocked
IS-No	NO	Inexperienced	(-)
IS-SP	YES	Inexperienced	Shocked

**Figure 2:**
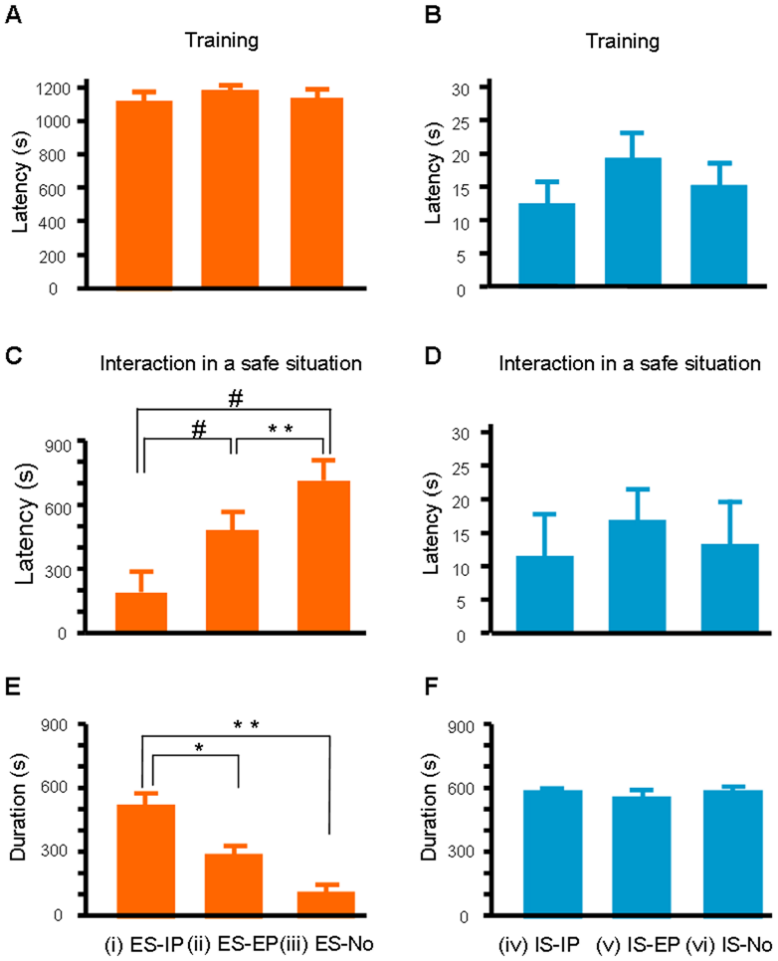
The effect of social interaction on avoidance behaviors in a safe situation. (A) Step-through latency (mean+s.e.m.) of the experienced subjects during the testing performed 24 h after shocking in the dark compartment of the experimental apparatus. (B) The step-through latency of the inexperienced subjects. (C) The latency of experienced subjects after interaction with inexperienced partners (ES-IP), after interaction with experienced partners (ES-EP), and after no interaction (ES-No). (D) The latency of inexperienced subjects under the three conditions (IS-IP, IS-EP, IS-No). (E-F) The duration of staying in the dark compartment. The number of subjects was ES-IP (n = 10$); ES-EP (n = 10); ES-No (n = 10); IS-IP (n = 9), IS-EP (n = 10$), IS-No (n = 9). $: Marked conditions were measured at the same time. The means±s.e.m. are represented as bars. The duration of one IS-IP subject was deleted due to the failure of measurement. (*, *p*<0.05, **, *p*<0.01, #, *p*<0.001)

One day after the preparation (day 2), we measured the step-through latency of both subjects of each pair individually. All of the experienced rats refrained from entering the dark compartment within 5 min (mean±s.e.m. = 1102+40 s), while inexperienced rats entered within 1 min (mean±s.e.m. = 15+2 s). The difference among the groups in experienced subjects (*p*>0.6, for all pairs, Figure 2A) was not significant. A similar result was found in inexperienced subjects (*p*>0.5, for all pairs, Figure 2B). The next day, we measured the latency with social interaction under the safe condition. We found that the latency of the ES-IP group was significantly shorter than that of the ES-EP (*p*<0.001, ES-IP vs. ES-EP) and ES-No (*p*<0.0001, ES-IP vs. ES-No) groups. Interestingly, the avoidance responses of rats in the ES-EP group was also shortened (*p*<0.01, ES-EP vs. ES-No, Figure 2C). The latencies of all three groups of inexperienced subjects, however, were not different from one another (*p*>0.8, for all pairs, see Figure 2D). Similar results were found in the staying duration in the dark compartment of both experienced and inexperienced subjects (Figure 2E–F).

### The effect of social interaction on avoidance behavior in a dangerous situation

**Figure 3:**
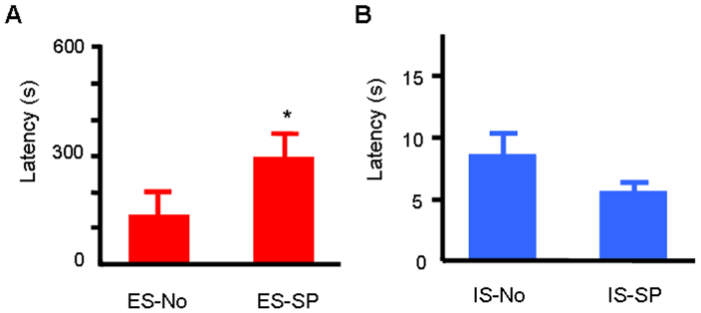
The effect of social interaction on avoidance behaviors of ES and IS in a dangerous situation. (A) Latency of the experienced subjects under an asocial condition (ES-No) and under a social condition (ES-SP). (B) Latency of the inexperienced subjects under a non-interactive condition (IS-No) and under an interactive condition (IS-SP). The numbers of experienced subjects (ES-No and ES-SP) and that of the inexperienced subjects (IS-No and IS-SP) were n = 16 and n = 12, respectively. * *p*<0.05.

The partners were given a foot shock stimulus during the retention time of the subjects, and we then compared the latency between asocial and social conditions. All the conditions tested are described in Table 1. This behavioral test was conducted using identical animals because the avoidance behavior of the experienced subjects can vary individually. First, we measured the subjects' basal avoidance without social interaction (ES-No, IS-No). The mean latency of ES-No was 123.6±19.4 (s), and that of the IS-No was 8.3±1.6 (s). There was a significant difference between the ES-No and IS-No groups (*p*<0.001). After a 30-min interval, the subjects were placed in the experimental setting again, where they interacted with shocked partners (ES-SP, IS-SP), and the latencies of the subjects were measured. The latency of the ES was significantly increased by the interaction with shocked partners (ES-No vs. ES-SP, *p*<0.05, Figure 3A). Not all, but some of them showed clearly prolonged retention. On the other hand, the avoidance behavior was not enhanced in inexperienced subjects at all. Their latency tended to decrease rather than increase (IS-No vs. IS-SP, *p* = 0.1, Figure 3B). These results indicate that the information from shocked partners had a facilitatory effect on avoidance in the experienced subjects.

## Discussion

In the current study, the behavioral influences of social interaction between two rats in a changing environment were systematically evaluated by focusing on the previous experience of passive avoidance learning. The major results were as follows: (1) learned avoidance behavior was inhibited by social interaction with neighboring partners, especially partners who had not learned avoidance behavior; (2) avoidance behavior of experienced rats was reinstated by shocked partners; (3) there were none of the inexperienced rats whose avoidance behavior was modified by any kind of partner. Taken together, these results indicate that previous learning is a crucial factor for the social enhancement or inhibition of avoidance in rats. Our findings suggest a view in which the prerequisites for the social transmission of avoidance may include previous learning experience of subjects as well as alarming social cues from others.

### Social interaction induces an inhibitory influence on the avoidance of experienced subjects in safe conditions

The experienced subjects were inhibited by the partners under the no-shock conditions. These inhibitory influences of social interaction were also found in learned aversion to a flavored food [23] and conditioned fearful response [24]–[25]. The results of this study present that the social interaction has the inhibitory effect also on the passive avoidance in rats. A new finding of the present study is that inhibitory influences depend on a partner's experience. The strength of inhibitory influence was much higher by inexperienced partners than by experienced partners. This suggests that the previous learning of partners has a specific role in the social modulation of avoidance. How do social partners affect avoidance behavior of other individuals? Some studies have shown that individual vigilance was depressed by increasing group size [26]–[27] or by shortening neighbor distance [28] in various animals. The depressed vigilance may prompt an inhibitory influence on avoidance. These effects can explain the inhibitory influence of experienced partners. The group effect cannot explain why the influence of inexperienced partners is higher than that of experienced partners. Inexperienced partners inhibited the avoidance more strongly than did experienced partners, even though the two rats were placed in a very limited space under the ES-EP conditions. Therefore, there are likely other mechanisms at work. One most likely reason why the effect was bigger with the inexperienced partners rather than the experienced partners is that the subjects followed the partners. Rats have been thought to have some high-order cognitive abilities such as imitation through observation of acting others [29]–[30] and causal reasoning [31]. Two other possibilities are: (1) the rats might imitate the behavior of inexperienced partners introduced to the dark compartment without awareness, and (2) the rats might expect extinction of the dangerous stimuli by inference from the partners' behavior. These two possibilities are formed by the independent effect of observation, and it would be necessary to examine this effect to know if these possibilities are feasible.

### Social interaction with shocked partners can induce a facilitatory influence on avoidance

As already mentioned, previous studies suggested that social transmission of avoidance does not occur in naïve rats [13]–[15]. The present results that the avoidance behavior of inexperienced subjects was not facilitated by social interaction under either no-shock conditions or shock conditions are consistent with the results of previous studies. However, we observed that social interaction facilitated avoidance in avoidance-experienced rats. A previous study also showed that conditioned fear was recovered by the presentation of shocked partners in Pavlovian conditioning [32]. Our results provide the possibility of social transmission of avoidance in an operant learning paradigm, that is, not only under Pavlovian conditioning but also operant conditioning. The present systematic experiments empirically showed the unexamined differences between avoidance-related adaptation of experienced rats and inexperienced rats under social environments.

### Social cues for the social transmission about avoidance in rats

Animals transmit various types of social cues, and those signals tell important information to other companions. The present results clearly demonstrate that social cues from a partner determine the contents of social transmission. Social cues emitted by partner rats can be categorized into two types according to the partners' situations regarding stimulus application. One category of social cue is accompanied by punishment or negative stimulus such as an electrical shock to individual animals. This type of social cue can be an announcement of an aversive situation or danger for others. Actually, for example, fish emit alarm substances when they are attacked by an enemy. Those substances are social cues that trigger avoidance in others [33]–[34]. Another category of social cue is accompanied by reward or positive stimulus such as food to individuals. That can be an announcement of a favorite situation or safety for others. The social transmission of food preference in rats [2] is an example.

What signals are important for the adaptation to a changing environment? In the present study we investigated the social transmission of information with environmental change from danger to safety and vice versa, and our results may help to answer the question. The experimental design allowed partners to have interaction with subjects. In Experiment 2, the partner was able to transmit sensory information including (1) alarming vocalization emitted when the partner was shocked, (2) smell or pheromone in excretion such as urine and feces, and (3) struggling motion. Shock or stress can induce alarming vocalization (ultrasonic vocalization) [35]–[36] and alarming odors [37] in rats. The timing of transmission varies according to the nature of the information. Vocalization and struggling motion tended to be emitted just after the partner was shocked, and then they faded within a few seconds. In contrast, odor information was emitted from the shocked partner after shocking, but it lasted a relatively long time. Therefore, one of those forms of sensory information or a combination of them may have acted as signals to announce danger. For an announcement of safety, the lack of a shock-induced reaction of partners may be an important signal. In social animals, avoiding or facilitating a behavior by many types of social cue effectively controls their adaptation to a changing environment.

### How does individual experience affect social transmission?

The present results demonstrate that social interaction affects experienced subjects' behavior but not inexperienced subjects' behavior, especially in a dangerously-changed situation (Figure 3). This indicates that there is an experience-dependence of social interaction in avoidance behavior and that previous individual experiences play an important role in social transmission. What is the importance of the learning experience in the processes of social transmission? There seem to be two possibilities, at least. First, individual experiences work to enhance the acquisition of information from other animals during observation. Some studies indicate aversive experiences enhance the sensitivity of animals with respect to the acquisition of information [38]–[40]. Getting information from others is the first step of social transmission. There is no doubt about the importance of the quality of getting information in social transmission. How can this explain the present results? By following this hypothesis, experienced subjects were affected by other partners because of the enhancement of previously gained sensitivity, but inexperienced subjects were not affected because their sensitivity level was not high enough. This interpretation can partially explain the present results, but it is difficult to explain all of the results with only this interpretation for the following reason. In this experiment none of the inexperienced subjects was affected by partners, although inexperienced subjects received similar social cues to those received by experienced subjects. Actually, a previous study has shown that inexperienced rats get information from other conspecifics showing fear responses [41]. This is inconsistent with the first hypothesis, but second hypothesis can explain that result.

A second possible reason for the importance of the learning experience in the processes of social transmission is that when getting social cues, individual experiences are recalled and help the receiver to associate individual experience with information from other conspecifics to plan the next appropriate action. This is another promising hypothesis. If avoidance-learning is recalled under the influence of a partner's cues, avoidance behavior will be quickly reacquired even after avoidance responses are extinct. Although there is no direct evidence that individual memory is recalled via another conspecific in rats, memory can be recalled by various associative stimuli. The neural mechanism where social cues are associated with individual experiences should be elucidated in the future. These two possible functions may support the notion of stages of social transmission.

Our results provide evidence that individual experience is one of the important factors for social enhancement or inhibition of avoidance behavior. It may be that we have little knowledge about the social transmission of avoidance because behavioral experiments focusing on individual experience are not so popular. Additional progress of the behavioral studies considering individual experience may facilitate the understanding of the neural mechanism for social learning through cooperation with researchers conducting neurological studies that have been revealing the neural mechanisms of various types of learning.

### Conclusion

In conclusion, we systematically investigated social influence on avoidance behavior under a situational change, focusing on the previous experience of rats. Throughout our experiments, the experienced subjects were influenced by experienced or inexperienced partners depending on changing experimental situations. The results suggest that rats can adapt their behaviors by utilizing both social interaction with a variety of types of partners and individual experiences in dynamically changed situations.
